# Conditional QTL Analysis and Fine Mapping for Thousand-Kernel Weight in Common Wheat

**DOI:** 10.3390/plants14121848

**Published:** 2025-06-16

**Authors:** Haoru Guo, Wei Liu, Geling Yan, Yifan Dong, Chongshuo Guan, Zhiyan Zhang, Changhao Zhao, Linxuan Xia, Da Zhu, Chunhua Zhao, Han Sun, Yongzhen Wu, Jianguo Wu, Ran Qin, Fa Cui

**Affiliations:** 1 Yantai Key Laboratory of Molecular Breeding for High-Yield and Stress-Resistant Crops and Efficient Cultivation, Modern Seed Industry and Green Planting & Breeding Research Center, College of Horticulture, Ludong University, Yantai 264025, China; 13642172805@163.com (H.G.); weiliushandong@163.com (W.L.); 15376256818@163.com (G.Y.); 19712075719@163.com (Y.D.); guanchongshuo@163.com (C.G.); 18660727968@163.com (Z.Z.); 18853915154@163.com (C.Z.); 19235348736@163.com (L.X.); 15506385653@163.com (D.Z.); sdauzch@126.com (C.Z.); sunhan@ldu.edu.cn (H.S.); yongzhenwu1204@163.com (Y.W.); 2 College of Horticultural Science, Zhejiang A&F University, Linan 311300, China; jianguowu@zafu.edu.cn

**Keywords:** wheat, thousand-kernel weight, conditional QTL, unconditional QTL, fine mapping

## Abstract

To elucidate the genetic basis of thousand-kernel weight (TKW) related to fundamental traits such as kernel length (KL), kernel width (KW), and kernel diameter ratio (KDR) at the individual quantitative trait loci (QTL) level, both unconditional QTL analysis and conditional QTL analysis for TKW were analyzed using a recombinant inbred line (RIL) population, along with a simplified physical map. A total of 37 unconditional QTLs and 34 conditional QTLs were identified. Six QTLs exhibited independent effects from individual traits (KL, KW, or KDR), while 18 QTLs showed common influences from two or three of these traits simultaneously. Additionally, 26 pairs of epistatically interacting QTLs involving 16 loci were detected. Subsequently, fine mapping of the stable and major-effect QTL *QTkw1B* was carried out using the derived near-isogenic lines (NILs), ultimately locating it within the interval of 698.15–700.19 Mb on chromosome 1B of the KN9204 genome. The conditional QTL analysis and genetic effect analysis based on NILs both indicated that the increase in TKW was primarily contributed by kernel length. The QTL identified in the present study through the combination of conditional and unconditional QTL mapping could increase the understanding of the genetic interrelationships between TKW and kernel size traits at the individual QTL level and provide a theoretical basis for subsequent candidate gene mining.

## 1. Introduction

Wheat (*Triticum aestivum* L., 2n = 42, AABBDD) is one of the most widely cultivated and geographically distributed food crops globally. With the global population projected to reach 9.1 billion by 2050, food demand is expected to surge by 50–100%, necessitating yield improvements in staple crops (http://www.fao.org). In wheat, spike number per plant (SNPP), kernel number per spike (KNPS), and thousand-kernel weight (TKW) are three key factors influencing wheat yield. Under the premise of stable SNPP and KNPS, the increase in kernel weight directly determines the rise in wheat yield [1]. TKW is a quantitative trait controlled by both major and minor effect genes, influenced by factors such as kernel length (KL), kernel width (KW), and kernel thickness (KT), with relatively low environmental sensitivity and high heritability [2]. Researchers have identified QTL associated with TKW on various chromosomes. For example, Guan et al. [3] located a TKW QTL on chromosome 4A, narrowing down the target region to approximately 6.5 Mb (677.11–683.61 Mb), where favorable alleles could increase TKW by 5.16–27.48%. Brinton et al. [4] used near-isogenic lines to identify and finely map a major *QTL-Qtkw-cb.5A* related to kernel weight on chromosome 5A, locating it within a 4.3 cM interval. Zhao et al. [5] identified a TKW locus *QTgw.caas-5B* on chromosome 5B, which was ultimately refined to a region of about 2.0 Mb between markers *Kasp_5B29* and *Kasp_5B31*, corresponding to the physical region of 49.6–51.6 Mb.

Traditional QTL studies primarily focus on uncovering associations between complex traits and genetic markers but often overlook the potential genetic correlations among these traits, and their genetic interrelationships have not been thoroughly evaluated. For example, studies have found that QTLs for TKW share common regions or identical QTLs with other kernel traits [6,7,8,9]. However, in these reports, QTL analyses were conducted separately based on phenotypic values of kernel traits, only revealing the correlations and interference among complex traits without evaluating their actual genetic relationships. Traditional QTL studies do not actually evaluate closely related traits and the genetic contribution to a single trait. By contrast, conditional QTL analysis allows for the assessment of the contribution of each trait to the complex trait. To address this issue, multi-variable conditional analysis was introduced for the study of complex traits and their component factors, providing an effective means of investigating genetic correlations between related traits at the individual QTL level [10]. Currently, this method is widely used for the analysis of closely related traits [11,12,13,14,15,16,17]. For TKW, Zhang et al. [13] analyzed the genetic relationships between wheat TKW and related traits such as KL and KW using both unconditional and conditional QTL methods, and the results showed 36 unconditional and conditional additive QTLs, which demonstrated that the effects of the 25 additive QTLs for TKW were either entirely or largely determined by KW, while another 25 TKW additive QTLs were either completely or largely influenced by KL. Conditional mapping can be useful for a better understanding of the interrelationship between the yield-contributing traits at the QTL level. Li et al. [18] combined conditional and unconditional mapping methods to investigate the correlation between TKW and kernel size, further illustrating that KL, KW, and KDR contribute to TKW to varying degrees. High genetic correlations between TKW and kernel traits also suggest high genetic control. Conditional QTL mapping for TKW dissects these relationships by comparing the results of unconditional and conditional QTL mapping: (a) QTLs with similar or equal effect in both two analyses, indicating that the TKW QTL is independent of the given trait; (b) QTL with significantly reduced or enhanced effects in both two analyses, suggesting that the TKW QTL is partially influenced by the related trait; (c) QTL detected in unconditional analysis but not in conditional analysis, implying that the TKW QTL is entirely controlled by the conditioning trait; (d) additional QTL detected in conditional analysis but not unconditional analysis, indicating that the expression of TKW QTL is completely suppressed by the conditioning trait, and the effects could only be identified by excluding the influence of the conditioning trait [5,11,17].

Complex traits in wheat may be also co-regulated by multiple interacting QTLs, where the functional expression of certain genes depends on specific allelic variants of other genes. Epistatic analysis helps elucidate polygenic networks and provides a theoretical foundation for crop genetic improvement. Li et al. [18] also revealed three pairs of QTLs with epistatic interactions for TKW, and all the epistatic interactions occurred between adjacent loci on chromosome 1A. Epistatic interactions between *QTkw1A.1-19* and *QTkw1A.1-25* had the most pronounced effect and accounted for 9.17% of phenotypic variance. Ma et al. [19] found epistatic interaction between the major QTL-*QYr.nwafu-6BL.2* and *QYrsnb.nwafu-2BL* for wheat stripe rust resistance, and *QYrsnb.nwafu-2BL* could accelerate the expression of *QYr.nwafu-6BL.2* to enhance resistance with epistatic interaction. Thirty pairwise QTLs with epistatic effects were identified in the trait of wheat awn length, in which the effects of *qAl-2A* and *eqAl-2A-1* were suppressed due to an interaction, resulting in a significant reduction in additive effects [20]. Spike shattering can cause severe grain yield loss in wheat. The cultivar Carberry contributed two major QTLs associated with spike shattering on chromosome arms 4BS and 5AL. In the epistatic QTL analysis, the interaction between the QTLs on chromosomes 4BS and 5AL was found to be the most consistent and synergistic, and this interaction reduced the expression of shattering [21].

The wheat genome is highly extensive. Although numerous major-effect QTLs for TKW have been identified, studies systematically investigating the relationships between TKW and its influencing factors (such as KL and KW) at individual QTL levels remain relatively scarce. Using conditional QTL and epistatic QTL analyses, we can more accurately select loci and individuals with excellent traits under specific environments and reveal synergistic or antagonistic roles of genes, so that we can provide more comprehensive genetic information for breeding. At the same time, in-depth analysis and gene mining of the main effective kernel weight loci can help to improve the breeding efficiency and thus increase the yield potential of wheat. In present study, a population of recombinant inbred lines (KJ-RILs) constructed from KN9204 and J411 was used for unconditional QTL and conditional QTL analysis as well as epistatic QTL analysis for TKW with a high-density physical map. The objectives here are to (1) identify unconditional QTLs for TKW in ten environments; (2) clarify the genetic relationship between TKW and KL, KW, and KDR at the individual QTL level using conditional QTL analysis; and (3) perform primary fine mapping based on the stable and major QTLs for TKW derived from the unconditional and conditional analysis and develop closely related molecular markers to analyze its genetic effects. This work lays a crucial foundation for identifying the target genes of TKW QTLs and provides a reference for improving varieties through marker-assisted selection and relevant fundamental research.

## 2. Results

### 2.1. Phenotypic Analysis of the Wheat RIL Population

**Figure 1:**
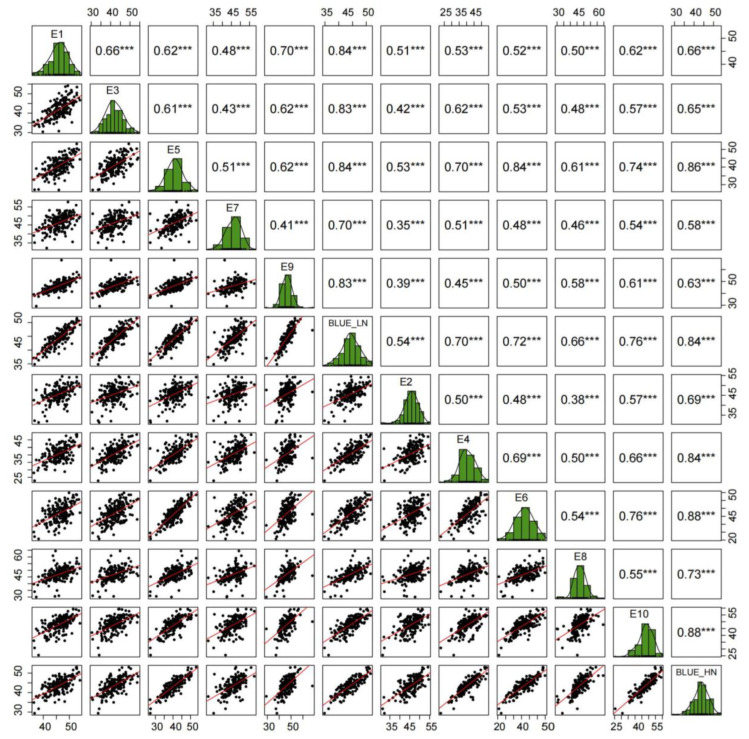
Frequency distribution and correlation analysis of TKW data in the KJ-RIL population in various environments. *** represents the significance level of *p* < 0.001.

To assess variance among materials, the TKW of the KJ-RIL population and their parents was examined in 10 environments. The mean TKW of the population ranged from 37.34 to 47.99 g across different environments, with a coefficient of variation (CV) spanning 7.46–13.75% (Table 1). The frequency distribution analysis of TKW in the KJ-RIL population under ten environments showed that the TKW exhibited a continuous distribution characteristic across multiple environments, with absolute values of skewness coefficients ranging from 0.05 to 0.49 and absolute values of kurtosis coefficients ranging from 0.07 to 2.03 (Table 1; Figure 1), indicating a typical quantitative trait controlled by multiple genes. QTL mapping can be conducted on the KJ-RILs using quantitative trait analysis methods. Additionally, significant correlations in TKW were observed across paired environments (Figure 1).

### 2.2. Unconditional QTL Analysis for TKW in the Wheat RIL Population

**Figure 2:**
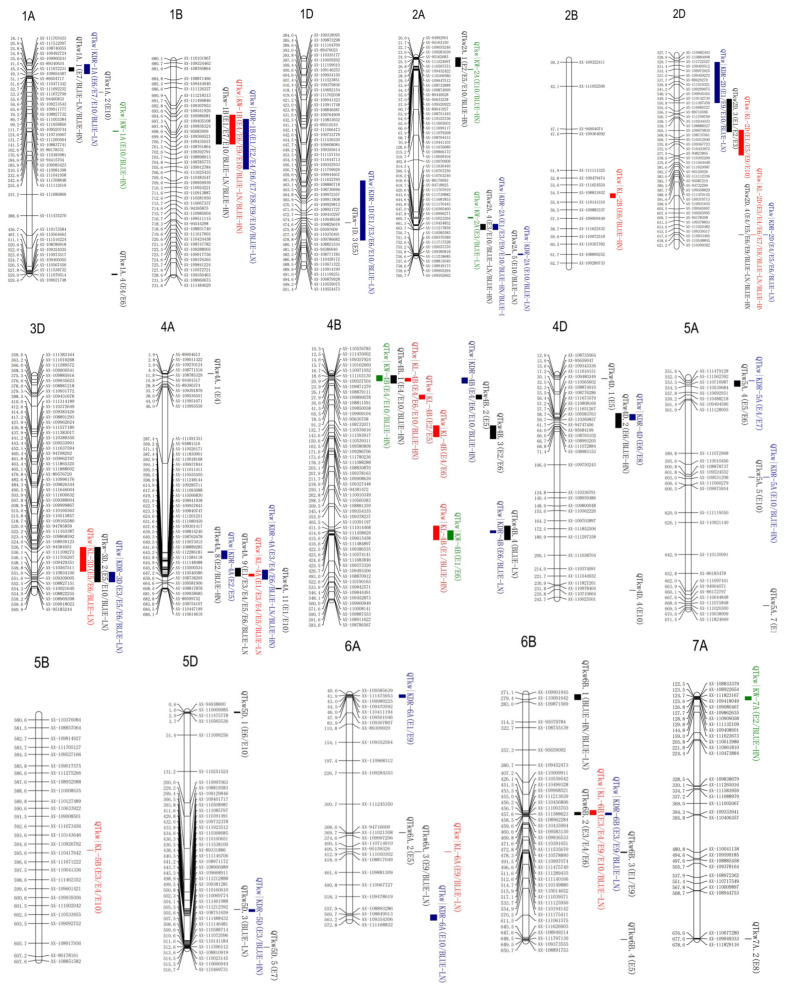
Chromosomal locations of conditional and unconditional QTL for kernel traits. The map markers are listed on the right side of the corresponding chromosomes. Physical locations of markers are indicated on the left side of the chromosomes. The combinations of letters and numbers at the top of each image represent the wheat different chromosomes.

Based on the previously constructed physical map and multi-environment kernel weight data from the 187 KJ-RILs, a total of 37 unconditional QTL effects TKW were detected across ten environments, as well as the BLUE (best linear unbiased estimates) values under low nitrogen (BLUE-LN) and high nitrogen (BLUE-LN) levels, respectively (Table 2; Figure 2). These QTL were mapped to 15 chromosomes: 1A, 1B, 1D, 2A, 2D, 3A, 3D, 4A, 4B, 4D, 5A, 5D, 6A, 6B, and 7A. These QTLs exhibited a range of LOD values from 2.52 to 24.23, explaining 2.39–22.43% of the phenotypic variation. Among these, 17 QTLs had positive effect alleles from KN9204, and 20 had negative effect alleles from J411. In addition, a total of 14 QTLs were located on chromosomes 1A, 1B, 2A, 2D, 4A, 4B, 6A, and 6B, with an average phenotypic variations explained (PVEs) index greater than 5% and detected in at least two datasets, which were recognized as the major stable-effect QTLs, including *QTkw1A.1*, *QTkw1B*, *QTkw2A.4*, *QTkw2D.3*, *QTkw2D.4*, *QTkw4A.4*, *QTkw4A.6*, *QTkw4A.8*, *QTkw4B.1*, *QTkw4B.3*, *QTkw6A.3*, *QTkw6B.1*, *QTkw6B.2*, and *QTkw6B.3*. Specifically, *QTkw1B* was detected in five datasets, with the PVE ranging from 2.73% to 9.50%, and *QTkw2A.4* was detected in three datasets, with a PVE of 2.66–9.26%. *QTkw4B.1* was detected in three datasets, with the PVE ranging from 4.51% to 22.43%, and *QTkw6B.2* was detected in three datasets, with a PVE of 5.38–10.12%. The additive effects of these QTLs indicated that favorable alleles were contributed by J411. Both *QTkw2D.4* and *QTkw4A*.6 were detected in six datasets, with PVEs of 6.40–13.47% and 3.76–9.76%, respectively.

### 2.3. Conditional QTL Analysis for TKW in KJ-RIL Population

To elucidate the genetic relationships between TKW and other kernel traits at the individual QTL level, conditional QTL analysis was performed for TKW across 12 sets data using KL, KW, and KDR as conditioning traits, respectively (Table 3; Figure 2). The changes in the proportion of PVE of one QTL between conditional and unconditional analyses were used to analyze the genetic relationships of TKW with KL, KW, and KDR. Comparing the results of unconditional and conditional QTL profiles leads to four outcomes: (a) QTL with similar or identical effects in both two analyses; (b) QTL with significantly reduced or enhanced effects in both two analyses; (c) QTL detected in unconditional analysis but not in conditional analysis; and (d) additional QTL detected in conditional analysis but not unconditional analysis.

The results showed that when conditioned on KL, 12 QTLs for TKW were detected, distributing across chromosomes 2B, 2D, 3D, 4A, 4B, 5B, 6A, and 6B, with PVEs of 2.46–33.55%. Among these, *QTkw2D.4* and *QTkw4A.6* had comparable PVEs between unconditional and conditional QTL analyses, indicating that this QTL did not influence TKW through its effect on KL. Eight QTLs, including *QTkw2D.3*, *QTkw3D.2*, *QTkw4B.1*, *QTkw4B.2*, *QTkw4B.3*, *QTkw4B.4*, *QTkw6A.3*, and *QTkw6B.2*, were detected in both conditional and unconditional analyses, with significantly higher or lower effect values, suggesting that these QTLs influenced TKW partially through their effects on KL. Eleven QTLs (*QTkw1A.1*, *QTkw1B*, *QTkw1D.3*, *QTkw2A.4*, *QTkw2A.5*, *QTkw4A.4*, *QTkw4D.2*, *QTkw5A.4*, *QTkw5A.5*, *QTkw5D.3*, and *QTkw7A.2*) could be detected in unconditional analysis but were undetectable in the conditional constraints of KL, suggesting that these QTLs were completely affected by KL. Additionally, *QTkw2B* and *QTkw5B.5* were only detected in the conditional QTL analysis, indicating that the expressions of these two QTLs were suppressed by KL.

When the effect of KW on TKW was excluded, seven QTLs for TKW were detected, distributing across chromosomes 1A, 1B, 2A, 4B, and 7A, with PVEs of 4.16–20.21%. An average PVE of 9.90% was detected for *QTkw1B*, which was significantly higher than that of its unconditioned QTL (5.55%). This phenomenon suggests that the additive effect of this QTL on TKW was partly derived from KW’s effect. In contrast, *QTkw4B.1* explained 7.1% of the phenotypic variation on average, which was significantly lower compared to that of the unconditional QTL (15.79%), again suggesting a partial effect of KW on this QTL. Furthermore, *QTkw1A.3*, *QTkw2A.2*, *QTkw2A.3*, and *QTkw7A.1* were only detected in the conditional QTL analysis, indicating that these QTL had an opposite additive effect on TKW and KW.

When TKW was conditioned on KDR, a total of 20 QTLs for TKW were detected. Among these, *QTkw1B*, *QTkw2A.4*, *QTkw4B.4*, *QTkw4D.2*, *QTkw5A.4*, and *QTkw5D.3* exhibited significantly higher PVEs compared to their corresponding unconditional QTL; *QTkw2D.3*, *QTkw2D.4*, *QTkw4B.1*, *QTkw5A.5*, *QTkw6B.2*, and *QTkw7A.2* showed significantly lower PVEs compared to their corresponding unconditional QTL, indicating they were partially effected by KDR. *QTkw1A.1*, *QTkw1D.3*, *QTkw2A.5*, *QTkw3D.2*, *QTkw4A.4*, and *QTkw4A.6* had PVEs which were statistically indistinguishable from their corresponding unconditional QTL. Additionally, *QTkw6A.1* and *QTkw6A.4* were only detected in the conditional QTL analysis, indicating that the expressions of these two QTLs were suppressed by KDR.

In summary, we identified (1) six QTLs exhibiting independent effects from individual traits (KL, KW, or KDR), (2) eighteen QTLs showing common influences from two or three of these traits simultaneously, and (3) eight additional QTLs detected in conditional analysis, indicating that the expression of these QTLs for TKW was completely suppressed by one of the traits (KL, KW, or KDR), which had opposite additive effects on TKW and kernel traits.

### 2.4. Epistatic QTL Analysis for TKW in the KJ-RIL Population

A total of 26 pairs of QTLs with epistatic effects for TKW were identified in the KJ-RILs. These epistatic QTLs explained 5.02% to 9.97% of the phenotypic variation in TKW and were distributed across chromosomes 1D, 2A, 2D, 3A, 3B, 3D, 4A, 4B, 5A, 5B, 5D, 6A, 6D, 7A, 7B, and 7D (Table S1). Most of the epistatic interactions occurring between adjacent loci on chromosomes 2A, 3A, and 4A. Two pairs of QTLs, *QTkw3A.3/QTkw4A.4* and *QTkw4A.4/QTkw5A.1*, showed the most significant epistatic interaction effects, explaining 9.29% and 9.97% of the phenotypic variation, respectively (Table 4).

For KL, 25 pairs of epistatic QTLs were identified, with epistatic interactions involving 13 loci on chromosomes 1D, 2A, 3A, 3B, 3D, 4A, 4B, 5A, 5B, 5D, 6A, 6D, and 7A (Table S2). Among them, *QKl3A.6/QKl5D.2* and *QKl4A.3/QK6D.6* accounted for 9.55 and 9.48% of KL variation, respectively (Table 4). For KW, 26 pairs of epistatic QTLs were detected, with 14 loci located on chromosomes 1A, 2A, 2B, 2D, 3A, 4A, 4B, 5A, 5B, 5D, 6A, 6B, 6D, and 7D (Table S3). The epistatic interaction between *QKw2B/QKw4A.1* explained 7.74% of the KW variation (Table 4). For KDR, 10 pairs of epistatic interactions involving 11 loci were detected, distributed across chromosomes 1B, 1D, 2A, 2D, 3A, 4A, 5B, 5D, 6D, 7B, and 7D (Table S4). The epistatic interaction between *QKdr5B.1/QKdr7B.2* was the most significant, accounting for 10.36% of the phenotypic variation (Table 4). These results suggested that epistatic interactions occurred between the loci on the same or different chromosomes.

### 2.5. Fine Mapping of QTkw1B in the NIL Population

**Figure 3:**
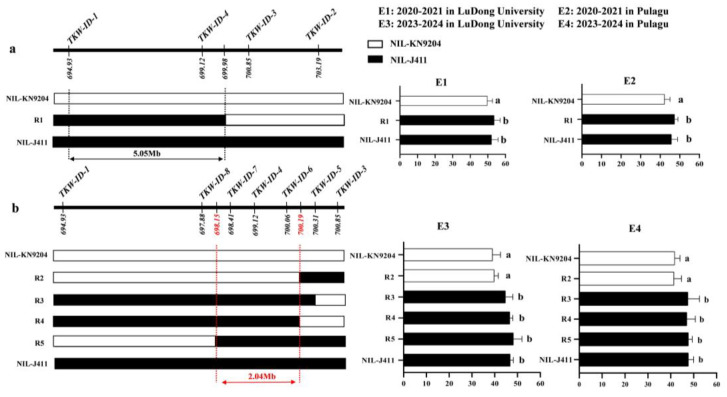
Fine mapping of *QTkw1B*. (**a**) Primary fine mapping of *QTkw1B* in 2020-2021. (**b**) Further fine mapping of *QTkw1B* in 2023-2024. On the left, genotype diagrams of different NILs in the target region are shown. The white bars represent the NIL-KN9204 genotype, which is consistent with the genotype of KN9204. The black bars represent the NIL-J411 genotype, which is consistent with the genotype of J411. The figure on the right shows the TKW values under different environments. The white columns indicate that the target segment genotypes of NILs are consistent with those of KN9204, while the black columns indicate that the target segment genotypes of NILs are consistent with those of J411. The mean value of TKW (±SD) is shown in each histogram. ANOVA analysis plus the LSD test was used for multiple comparison, and a shared letter within groups indicated no significant differences in TKW between NILs at the level of *p* < 0.05.

In this study, a stable and major *QTkw1B* was identified, and the conditional QTL analysis showed that the effect of *QTkw1B* was completely affected by KL and partially affected by KW and KDR. Based on these findings, we aim to further narrow down the target interval of *QTkw1B* through fine mapping. Using the developed InDel markers to genotype a secondary mapping population derived from self-pollination of the residual heterozygous line of F_8_ KJ-RILs, homozygous NILs (NIL-KN9204 and NIL-J411) and five key recombinants (R1 to R5) were identified. Multi-environmental TKW phenotyping of the above materials in conjunction with genotypic data and the fine mapping work of *QTkw1B* were conducted. Firstly, homozygous NILs (NIL-KN9204 and NIL-J411) and one recombinant R1 were obtained in 2020–2021 (Figure 3). Analysis of the phenotypic data showed that the TKW of both R1 and NIL-J411 was significantly higher than that of NIL-KN9204 in both E1 and E2 environments, suggesting that *QTkw1B* was located in the physical interval of 694.93–699.98 Mb of the KN9204 genome flanked by markers of *TKW-ID-1* and *TKW-ID-3.* Subsequently, we designed four new molecular markers (*TKW-ID-5* to *TKW-ID-8*) within the target interval of *QTkw1B* and genotyped the expanded secondary segregating population, and four new key recombinants were screened, named R2, R3, R4, and R5, respectively (Figure 3). The results showed that the TKW of R3, R4, R5, and NIL-J411 was significantly higher than that of R2 and NIL-KN9204 in both E3 and E4 environments. In summary, *QTkw1B* was finally classified within a physical interval of 2.04 Mb.

### 2.6. Genetic Effect Analysis of QTkw1B on Kernel and Yield-Related Traits

**Figure 4:**
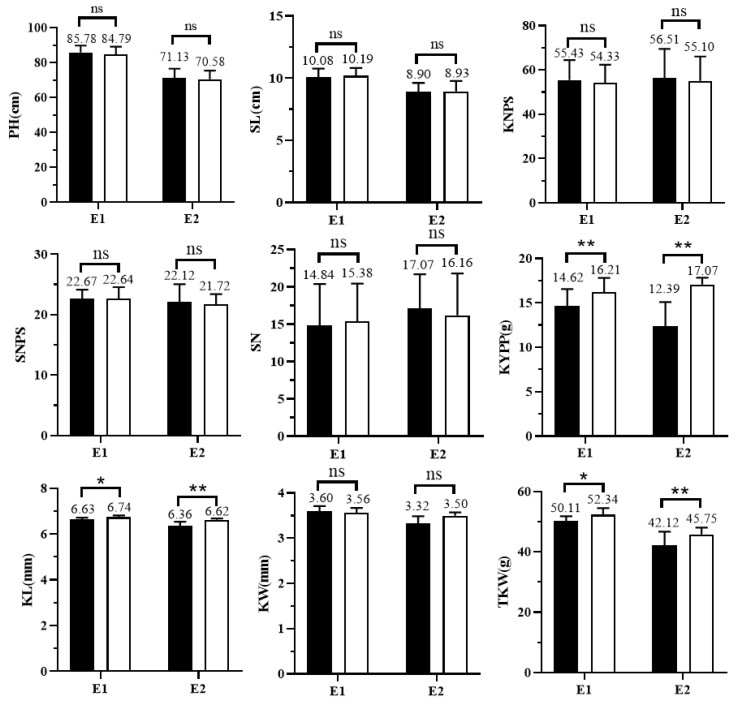
Genetic effect analysis of *QTkw1B* on wheat kernel and yield-related traits based on NIL pairs. E1, Ludong University experimental field (2020–2021); E2, Pulagu experimental field, Yantai (2020–2021). PH: plant height; SL: spike length; KNPS: kernel number per spike; SNPS: spikelet number per spike; SN: spike number; KYPP: kernel yield per plant; KL: kernel length; KW: kernel width; TKW: thousand-kernel weight. ** represents the significance level of *p* < 0.01. * represents the significance level of *p* < 0.05. ns represents no significance.

To determine the genetic effects of *QTkw1B* on yield-related traits, two pairs of NILs, NIL-KN9204 and NIL-J411 of *QTkw1B*, were phenotyped in multiple environments. The results showed that TKW, KL, and KYPP were significantly higher in NIL-J411 than those in NIL-KN9204 in both E1 and E2 environments, with average increases of 9.81%, 2.9%, and 24.3%, respectively (Figure 4). However, the allele of J411 had no significant effect on KW, KNPS, SNPS, SN, PH, and SL. The above results indicated that *QTkw1B* could increase the TKW by increasing the KL, thus realizing a significant increase in KYPP, while it did not have any significant effect on other yield-related traits, such as KW, SNPS, and KNPS.

## 3. Discussion and Conclusions

TKW is one of the key factors influencing wheat kernel yield. The complex genetic relationships between TKW and other kernel traits in unconditional analyses are largely unexplored. This study conducted a comprehensive unconditional and conditional QTL analysis, as well as an epistatic QTL analysis, to investigate the genetic basis of TKW and the interrelationships of TKW with KL, KW, and KDR. The genetic component effects of these kernel-related traits were identified. Compared to previous studies that focused only on the additive effect of individual QTLs, this research provides insights into the genetic basis and relationships between two or more closely related traits at the single QTL level [22,23,24,25].

Components of variance were analyzed for all traits. All of these traits showed significant *G*×*E* interaction effects (Table S6). The heritability of TKW (*h*^2^*B* = 0.32) indicates strong genetic control. Of the thirty-one unconditional QTLs detected for TKW, six QTLs were independent of KDR, two QTLs were independent of KL, and no QTLs were identified independent of KW (Table 3). This result was consistent with the genetic correlation between TKW and kernel traits (Table S7), which showed a highly significant positive correlation with KL (0.469) and KW (0.806) but a significant negative correlation with KDR (−0.513).

Stable and major-effect QTLs identified by unconditional QTL analysis, such as *QTkw1B* and *QTkw2D.4*, explain 2.73–9.50% and 6.40–13.47% of the phenotypic variation to TKW, respectively, indicating they were likely to be stable major-effect genes (Table 2). These findings were consistent with those of McIntyre et al. [26], Meng et al. [27], and Wang et al. [28]. Based on the results of conditional QTL analysis, *QTkw1B* was fully affected by KL and partially contributed by KW and KDR. Additionally, we found that *QTkw2A.4*, *QTkw4D.2*, *QTkw5A.4*, *QTkw5A.5*, *QTkw5D.3*, and *QTkw7A.2* were fully contributed by KL and KW, and partially by KDR (Table 3). *QTkw2D.3* and *QTkw6B.2* were primarily dependent on KW changes, with partial dependence on KL and KDR. Five additional QTL (*QTkw1A.4*, *QTkw2A.1*, *QTkw4A.8*, *QTkw6B.1*, and *QTkw6B.3*) for TKW in conditional analysis were detected (Table 3). One possible explanation for these additional QTLs is that they are genes with very small genetic effects and were undetected by unconditional analysis, but they were identified when excluding the influence of the conditioning trait, indicating their expressions were completely suppressed by the conditioning kernel trait, and that the additional QTL has an opposite additive effect on TKW and the conditioning trait. Wheat kernel weight was a quantitative trait controlled by multiple genes, and the results of this study showed that the TKW QTLs were contributed by KL, KW, and KDR to varying degrees, with KW being the most significant at the QTL level. This was consistent with the results of previous research that TKW had highly significant positive correlations with KL and KW, with the correlation coefficients being KW > KL [6,9,29,30]. Overall, these fundings laid an important foundation for the study of genetic regulation mechanism for TKW candidate genes in the future.

Epistatic interactions are another important factor in understanding the genetic mechanisms of complex quantitative traits [19]. Although epistatic effects may not be significant for the target trait, they can influence the identification of individual QTL and the efficiency and accuracy of marker-assisted breeding [31,32]. In this study, a total of 26 pairs of epistatic interactions were detected in TKW, which was also detected at different locations on the same chromosome or between different chromosomes. For example, *QTkw2D.1/QTkw2D.2* and *QTkw4B.4/QTkw4B.5* belonged to the QTL detected at different locations on the same chromosome, explaining 5.02% and 5.45% of the phenotypic variation, respectively (Table S1). Meanwhile, *QTkw3A.3* and *QTkw4A.4* as well as *QTkw4A.4* and *QTkw5A.1* belonged to interactions between different chromosomes and explained 9.29% and 9.97% of the phenotypic variation in TKW, respectively. On the other hand, although some QTLs do not have additive effects on TKW when present alone, they may indirectly affect TKW through epistatic effects and may be used as modifiers that activate or alter the function of other loci [33]. For the analysis of multiple genetic traits, the number of potential epistatic interactions effects is very large and deserves to be further investigated in wheat breeding research [6,9].

With the discovery of more QTLs for TKW in wheat, more QTLs will be finely mapped and candidate genes will be further identified. Song et al. finely mapped *QTKW.caas-5DL* using a secondary population derived from 15 heterozygous recombinants, and the QTL was located within a physical interval of approximately 3.9 Mb (409.9–413.8 Mb) on chromosome 5D based on the Chinese Spring reference genome [34]. Zhao et al. identified a major QTL, *QTgw.caas-5B*, which was delimited to an interval (49.6–51.6 Mb) of approximately 2.0 Mb flanked by the markers *Kasp_5B29* and *Kasp_5B31* by using 12 heterozygous recombinant plants [35]. Nezhad et al. mapped a TKW QTL near 565.99 Mb on chromosome 1B [36]; Cao et al. mapped a TKW QTL between 648.1 Mb and 652.1 Mb on chromosome 1B [37]. Comparative analysis revealed that the QTL mapped on chromosome 1B in these studies do not overlap with the interval of *QTkw1B* identified in this study. Liu et al. detected a TKW-related locus between markers *AX-109437338* and *AX-109032077*, which overlaps with the *QTkw1B* interval identified in the current study, but it has not been finely mapped [38]. Notably, we found the kernel weight QTL *QTkw1B* sharing the same localization interval (2.04 Mb) as the kernel length QTL *qKl-1BL* in our previous study, because the localization interval now is still large, and the two QTLs may be controlled by the same one gene or different genes [39]; however, in the conditional analysis, *QTkw1B* was undetectable when KL was excluded, suggesting that these QTLs were completely affected by KL, even though they was also partially affected by KW. This study indicates in another way that *QTkw1B* and *qKl-1BL* might be controlled by same one gene. In addition, the genetic analysis result of *QTkw1B* NILs showed that the increase in TKW was caused by KL but not KW (Table 3). Further narrowing of the target interval will help identify candidate genes for wheat kernel weight.

In this study, both unconditional and conditional analyses of wheat TKW were conducted using a simplified physical map, and on the basis of this result, we further narrowed down the *QTkw1B* interval to an interval of about 2.04 Mb using different NILs and key recombinants. Additionally, the genetic mechanism underlying *QTkw1B*’s regulation of TKW and other yield-related traits was elucidated using NIL material. The results collectively indicate that TKW is primarily determined by KL, and both traits are likely controlled by the same genetic locus. These findings have important theoretical and practical implications for breeding high-yielding and high-quality wheat varieties.

## 4. Materials and Methods

### 4.1. Plant Materials and Field Trials

In this study, KN9204 and J411 were selected as parents for the cross to produce the recombinant inbred line populations (KJ-RILs, KJ001-KJ187) using Single Seed Descent (SSD) method. The residual heterozygous lines (RHLs) in the target region for *QTkw1B* were screened in F_8_ KJ-RILs. By self-crossing the RHLs, the secondary mapping population was constructed, and then the NIL pairs as well as key recombinants were screened by molecular markers in the target region.

The parents and the population of KJ-RILs [39,40,41,42] were phenotyped at various periods of wheat planting, in 2011–2012 in Shijiazhuang in a low-nitrogen (LN) environment (E1), in 2011–2012 in Shijiazhuang in a high-nitrogen (HN) environment (E2), 2012–2013 in Shijiazhuang in an LN environment (E3), 2012–2013 in Shijiazhuang in an HN environment (E4), 2012–2013 in Beijing in an LN environment (E5), 2012–2013 in Beijing in an HN environment (E6), 2012–2013 in Xinxiang in an LN environment (E7), 2012–2013 in Xinxiang in an HN environment (E8), 2013–2014 in Shijiazhuang in an LN environment (E9), and 2013–2014 in Shijiazhuang in an HN environment (E10). In HN treatment, each HN plot received 300 kg ha^−1^ of diamine phosphate and 150 kg ha^−1^ of urea before sowing, followed by an additional 150 kg ha^−1^ of urea applied at the elongation stage annually. In contrast, In LN treatment, LN plots remained nitrogen-deficient, receiving no N fertilizer throughout the entire growing season. The field design and planting arrangement were described in previous studies [39,40].

The NIL pairs and the recombinant lines were planted in four environments, including the Ludong University experimental field and the Yantai Pulagu experimental field in 2020–2021 and 2023–2024, which were used for fine mapping of *QTkw1B* and its genetic effect on yield-related traits. The field management followed conventional farming practices.

### 4.2. Experimental Design and Phenotypic Evaluation

For KJ-RILs and NIL pairs, traits such as KL, KW, KDR, and TKW were determined using the TPKZ-3 intelligent seed test and analysis system (Zhejiang Top Cloud-agri technology, Hangzhou, China). Additionally, for NIL pairs, other yield-related traits such as PH, SL, KNPS, SNPS, SN, and KYPP were also measured. Each trait was evaluated from the average of the main tillers of the examined plants, and at least 15 individual plants with the same genotypes were selected randomly for phenotypic evaluation. All data were obtained from the averages of three replicates.

### 4.3. Genetic Map Construction and QTL Detection

We used a pre-obtained high-density genetic linkage map of wheat based on wheat 660K microarray genotype data from the KJ-RIL population [42]. The SNP flanking sequences were compared and analyzed using local BLAST (ncbi-blast-2.16.0+-win64.exe, https://ftp.ncbi.nlm.nih.gov/blast/executables/blast+/LATEST/) to obtain physical location information based on each SNP in the KN9204 genome [43]. A simplified physical map containing 7141 markers was obtained by de-redundancy analysis. Using the TKW data of the KJ-RIL population in ten environments, as well as the corresponding BLUE values, together with the physical map of KJ-RILs, a complete interval mapping method was performed by using softvare IciMapping 4.2 (https://www.isbreeding.net) to map the thousand kernels, with setting PIN = 0.001, Step = 1 cM, and LOD ≥ 2.5.

The conditional phenotypic values (TKW|KL, TKW|KW, and TKW|KDR), conditional on kernel length, width, and kernel size ratio, were calculated using QGAStation 2.0 [18]. Conditional QTL for conditional phenotypic values was analyzed by using IciMapping 4.2 with settings of PIN = 0.001, Step = 1 cM, and LOD ≥ 2.5. The conditional phenotypic value (T1|T2) represents trait T1 conditioned on trait T2. In the conditional QTL analysis, a T1 conditional QTL conditioned on T2 was considered to be unrelated to T2 if it had similar additive effect values and LOD values as a T1 unconditional QTL. The naming of the locus-dependent QTL followed the common international nomenclature (https://shigen.nig.ac.jp/wheat/komugi/genes/symbolClassList.jsp). For QTLs with epistatic effects, the walking speed chosen for all QTLs was 5.0 cM, and the *p* value inclusion threshold was 0.001. The threshold of the LOD score was manually set as 5.0. Physical locations less than 30 Mb apart were considered to be one locus. A QTL with an LOD score ≥ 2.5 and mean phenotypic variation explained (PVE) ≥ 5% was defined as a major QTL, and one showed significance in at least two environments.

### 4.4. Marker Development and Genotype Identification

A sequence comparative analysis between the highly assembled KN9204 genome and resequencing data of J411 was performed [43], and the InDels and SNPs in the target region were obtained. Polymorphic molecular markers were designed based on InDels (≥5 bp) by using PrimerServer in WheatOmics 1.0 (http://202.194.139.32/PrimerServer/). Genomic DNA was isolated using the cetyltrimethylammonium bromide (CTAB) protocol [44,45], followed by polymerase chain reaction (PCR) amplification and subsequent electrophoretic separation on 8% non-denaturing polyacrylamide gels, with visualization accomplished through silver staining. RHLs, NIL pairs, and recombinants were identified through these steps to finely map *QTkw1B*.

### 4.5. Data Analysis

Basic statistical analysis for the phenotypic data in the KJ-RIL populations was implemented by the software SPSS13.0 (SPSS, Chicago, IL, USA). The best linear unbiased estimation (BLUE) values for KL, KW, KDR, and TKW of the 188 KJ-RILs under high- and low-nitrogen environments were calculated, respectively, by using a linear mixed model with genotype as a fixed effect and environment (or environment × nitrogen level) as a random effect in the software of QGAStation 2.0.

The model can be expressed as follows:

$$Y_{ijk}=\mu +G_{i}+E_{j}+{\left(G\times E\right)}_{ij}+\epsilon_{ijk}$$

where *Y_ijk_* is the phenotypic value of the *i-th* genotype in the *j-th* environment for the *k-th* replicate, *μ* is the overall mean, *G_i_* is the fixed effect of genotype, *E_j_* is the random effect of environment, (*G*×*E*)*_ij_* is the interaction term, and *ϵ_ijk_* is the residual error.

We predicted genotypic values of each recombinant inbred line (RIL) in ten environments, i.e., the genetic predictors. Based on these datasets, genetic correlation analysis was conducted using SPSS13.0 software. To estimate the broad-sense heritability (*h_B_*^2^) of the corresponding traits and the genotype×environment interaction effects, ANOVA was performed using QGAStation 2.0 [46]. The *h_B_*^2^ values were calculated using the formula *h_B_*^2^ = V_G_/V_P_, where V_G_ and V_P_ are the genetic variance and phenotypic variance, respectively.

## Figures and Tables

**Table 1:** Analysis of phenotypic data of parents and KJ-RIL populations in different environments.

Traits	Env	Parents		RIL Population						
		KN9204	J411	Min	Max	Mean	SD	SK	KU	CV (%)
TKW (g)	E1	43.41 ± 0.05	45.18 ± 1.38	36.38	52.94	45.92	3.43	−0.35	−0.07	7.46
	E2	45.31 ± 1.56	40.94 ± 0.02	34.50	54.55	45.87	3.46	−0.25	0.39	7.55
	E3	40.17 ± 1.90	42.46 ± 4.60	33.03	51.84	41.89	4.10	0.12	−0.46	9.79
	E4	38.71 ± 1.20	41.83 ± 1.72 **	30.14	53.56	41.09	4.78	0.33	−0.26	11.63
	E5	39.02 ± 4.23	39.89 ± 1.36	30.52	50.44	40.99	4.30	0.08	−0.29	10.49
	E6	34.85 ± 2.98	34.65 ± 3.50	27.09	49.82	37.34	5.13	0.20	−0.61	13.75
	E7	47.02 ± 0.74	47.5 ± 1.41	35.16	55.95	46.19	3.94	−0.32	−0.20	8.53
	E8	46.33 ± 2.25	46.83 ± 2.64	30.04	60.91	46.98	4.65	−0.49	2.03	9.90
	E9	-	-	36.36	57.86	46.39	3.92	0.05	−0.17	8.45
	E10	-	-	35.43	59.28	47.99	5.10	−0.37	−0.14	10.62

SD, standard deviation; SK, skewness; KU, kurtosis; CV, coefficient of variation. ** represents the significance level of *p* < 0.01.

**Table 2:** Results of unconditional QTL analysis for TKW in the KJ-RIL population.

Unconditional QTL	Environment	Chromosome	Position (Mb)	Marker Interval	LOD	PVE(%)	Add
***QTkw1A.1***	E7/BLUE-LN/BLUE-HN	1A	35.13–51.13	AX-109492724-AX-110471342	4.94/3.55/2.88	8.52/4.02/3.34 (**5.29**)	1.34/0.64/0.70
*QTkw1A.2*	E10	1A	90.13	AX-111003384-AX-111659850	4.05	3.13	1.04
*QTkw1A.4*	E4/E6	1A	526.13	AX-109400055-AX-110567456	3.48/4.46	4.76/5.07 (4.91)	1.16/1.33
***QTkw1B***	BLUE-LN/E1/E7/E10/BLUE-HN	1B	693.16–700.16	AX-109307953-AX-109435037	2.72/5.51/2.79/7.51/3.96	2.73/9.50/4.80/5.74/4.96 **(5.55)**	−0.52/−1.06/−1.00/−1.41/−0.85
*QTkw1D.3*	E5	1D	477.13	AX-95097609-AX-110376491	3.04	3.43	−0.92
*QTkw2A.1*	E2/BLUE-HN/E5/E10	2A	21.93–55.93	AX-95163150-AX-95633238	2.52/3.98/4.04/3.51	4.64/ 5.28/4.08/2.52 (4.13)	−0.97/−0.88/−1.00/−0.93
***QTkw2A.4***	E3/E10/BLUE-LN	2A	641.93–662.93	AX-111193777-AX-111217859	5.99/3.76/6.42	9.26/2.66/6.95 **(6.29)**	−1.41/−0.96/−0.84
*QTkw2A.5*	E10/BLUE-LN	2A	754.93–755.93	AX-111717528-AX-111218685	7.30/2.79	5.56/2.66 (4.11)	1.39/0.52
***QTkw2D.3***	E3/E2/E1	2D	548.75–565.75	AX-109945455-AX-108737061	8.18/3.86/4.81	12.63/5.44/8.32 **(8.80)**	1.65/1.05/0.99
***QTkw2D.4***	E5/E9/BLUE-LN/E4/E6/BLUE-HN	2D	618.75	AX-110548845-AX-110399362	6.64/4.74/10.26/9.46/6.12/7.20	6.40/9.06/11.48/13.47/6.91/8.91 **(9.37)**	1.26/1.33/1.08/1.96/1.56/1.14
*QTkw3A.5*	E2	3A	726.76	AX-110026070-AX-10 9307603	2.83	4.46	−0.95
*QTkw3D.2*	E5/E10/BLUE-LN	3D	522.08–555.08	AX-94785859-AX-108969398	3.80/4.44/4.06	3.52/3.22/4.25 (3.66)	0.93/1.05/0.66
*QTkw4A.1*	E4	4A	2.91	AX-109011322-AX-109270124	2.64	3.27	−0.97
***QTkw4A.4***	E2/BLUE-HN	4A	567.91–568.91	AX-111591351-AX-94881516	6.86/3.10	11.23/3.68 **(7.46)**	1.50/0.73
***QTkw4A.6***	E1/E4/BLUE-LN/E3/E6/E5	4A	618.91–644.91	AX-111693688-AX-111148488	3.08/5.93/7.89/4.27/3.48/9.79	5.22/8.20/8.75/6.32/3.76/9.76 **(7.00)**	0.79/1.53/0.94/1.17/1.15/1.55
***QTkw4A.8***	E1/E10	4A	682.91–684.91	AX-89599752-AX-110614619	4.01/4.48	6.81/3.25 **(5.03)**	0.90/1.06
***QTkw4B.1***	E4/BLUE-HN/E10	4B	23.70–30.70	AX-111148234-AX-109284839	3.42/14.79/24.23	4.51/20.43/22.43 **(15.79)**	−1.14/−1.73/−2.79
*QTkw4B.2*	E5	4B	66.70	AX-109909104-AX-110066172	14.37	15.24	−1.94
***QTkw4B.3***	E6/E2	4B	173.70–178.70	AX-111780236-AX-110022862	16.95/3.68	21.82/5.16 **(13.49)**	−2.78/−1.02
*QTkw4B.4*	BLUE-LN	4B	423.70	AX-110574141-AX-111683846	5.23	5.43	−0.74
*QTkw4B.6*	E4	4B	591.70	AX-108911622-AX-108731280	3.90	5.12	−1.21
*QTkw4D.1*	E5	4D	16.53	AX-109343336-AX-111616151	2.84	3.04	0.86
*QTkw4D.2*	BLUE-HN/E6	4D	52.53–67.53	AX-108874010-AX-111072884	2.89/3.99	3.34/4.34 (3.84)	0.70/1.23
*QTkw4D.3*	E10	4D	236.53	AX-110710664-AX-110025501	6.13	4.54	1.25
*QTkw5A.4*	E5/E6	5A	551.67–554.67	AX-111479128-AX-110692951	2.80/3.59	2.57/3.92 (3.25)	−0.80/−1.17
*QTkw5A.5*	E10	5A	602.67	AX-109524552-AX-109031208	13.20	10.59	−1.91
*QTkw5A.7*	E10	5A	669.67	AX-111020300-AX-110038009	3.35	2.39	0.92
*QTkw5D.1*	E10/E6	5D	0.61–2.62	AX-94938800-AX-111475778	2.77/2.71	1.94/3.02 (2.48)	0.83/1.04
*QTkw5D.3*	BLUE-LN	5D	431.62	AX-110391491-AX-109732378	2.88	2.90	−0.54
*QTkw5D.5*	E7	5D	513.62	AX-108810919-AX-110023145	3.70	6.24	1.14
*QTkw6A.2*	E5	6A	368.01	AX-109086661-AX-94716600	6.52	6.24	−1.27
***QTkw6A.3***	E9/BLUE-LN	6A	413.01	AX-111050302-AX-108817049	3.33/5.82	6.21/6.09 **(6.15)**	−1.13/−0.81
***QTkw6B.1***	BLUE-HN/BLUE-LN	6B	271.76–279.76	AX-109901945-AX-109871569	4.41/7.43	5.22/7.96 **(6.59)**	−0.87/−0.89
***QTkw6B.2***	E4/E3/E6	6B	453.76–457.76	AX-109968521-AX-108962284	6.50/6.68/4.88	8.90/10.12/5.38 **(8.13)**	−1.59/−1.48/−1.37
***QTkw6B.3***	E1/E9	6B	514.76–515.76	AX-110149880-AX-110014652	4.19/3.80	7.03/7.11 **(7.07)**	−0.91/−1.17
*QTkw6B.4*	E5	6B	648.76	AX-111797130-AX-109373555	5.21	4.90	−1.10
*QTkw7A.2*	E8	7A	677.95	AX-109949333-AX-111829116	3.76	9.23	1.43

Physical locations less than 30 Mb apart was considered to be one locus. E1, E2, etc., indicate the environments in which the QTLs were detected. E1, E2: low and high nitrogen conditions in Shijiazhuang during 2011–2012. E3, E4: low and high nitrogen conditions in Shijiazhuang during 2012–2013. E5, E6: low and high nitrogen conditions in Beijing during 2012–2013. E7, E8: low and high nitrogen conditions in Xinxiang during 2012–2013. E9, E10: low and high nitrogen conditions in Shijiazhuang during 2013–2014. The same applies to the following. QTLs with average PVE values greater than 5 under multiple environments are shown in bold, and the numbers in “()” represent the calculated average PVE values.

**Table 3:** Results of conditional QTL analysis for TKW in the KJ-RIL population.

QTL	Marker Interval	Position (Mb)	Unconditional QTL		Conditional QTL					
					TKW|KL		TKW|KW		TKW|KDR	
			Environment	PVE (%)	Environment	PVE (%)	Environment	PVE (%)	Environment	PVE (%)
*QTkw1A.1*	AX-109310335-AX-111672231	22.13–45.50	E7/BLUE-LN/BLUE-HN	8.52/4.02/3.34 (5.29)					E10/E7/E6/BLUE-LN	3.61/8.56/3.95/2.96 **a** (4.77)
*QTkw1A.3*	AX-110717317-AX-109083423	184.13–185.13					E10/BLUE-HN	4.16/13.91 (9.03) **d**		
*QTkw1A.4*	AX-109400055-AX-110567456	524.66–526.52	E4/E6	4.75/5.07 (4.91) **c**						
*QTkw1B*	AX-109307953-AX-111625435	693.16–706.16	BLUE-LN/E1/E7/E10/BLUE-HN	2.73/9.50/4.80/5.74/4.96 (5.55)			E6/E9/BLUE-LN/E4/E10/BLUE-HN	4.53/8.41/15.43/10.27/12.14/8.63 **b** (9.90)	E2/E6/E1/BLUE-LN/E9/E7/E4/E8/E10/BLUE-HN	5.23/5.61/12.31/8.42/5.95/6.53/8.02/5.61/6.65/4.97 **b** (6.93)
*QTkw1D.3*	AX-89693771-AX-110671322	453.13–487.13	E5	3.43					E10/E6/E3/BLUE-LN/E1	3.49/3.68/5.54/2.94/4.60 **a** (4.05)
*QTkw2A.1*	AX-95163150-AX-95633238	21.93–55.93	E2/BLUE-HN/E5/E10	2.52/3.98/4.04/3.51 **c** (3.51)						
*QTkw2A.2*	AX-111605603-AX-111079268	65.93–66.93					BLUE-HN/E10	4.53/5.36 (4.95) **d**		
*QTkw2A.3*	AX-86179693-AX-94718925	616.55–619.68					E3/BLUE-LN	18.31/6.63 (12.47) **d**		
*QTkw2A.4*	AX-111193777-AX-111217859	641.93–662.93	E3/E10/BLUE-LN	9.26/2.66/6.95 (6.29)					E3/E1/E9/E10/BLUE-HN/BLUE-LN	13.31/6.31/5.66/8.36/4.57/11.13 **b** (8.22)
*QTkw2A.5*	AX-111717528-AX-111218685	752.58–756.56	E10/BLUE-LN	5.56/2.66(4.11)					BLUE-LN/E10	3.20/3.64 **a** (3.42)
*QTkw2B*	AX-108831932-AX-110141316	54.55–55.57			E6/BLUE-HN	4.82/4.13(4.48) **d**				
*QTkw2D.3*	AX-111722527-AX-111497459	529.75–550.75	E3/E2/E1	12.63/5.44/8.32 (8.80)	E1/E9/E10/E5	8.18/9.95/3.25/3.56 **b** (6.24)			E1/E10/BLUE-LN/E9	7.58/2.50/7.31/6.69 **b** (6.02)
*QTkw2D.4*	AX-110548845-AX-110399362	618.5–621.17	E5/E9/BLUE-LN/E4/E6/BLUE-HN	6.40/9.06/11.48/13.47/6.91/8.91 (9.37)	E3/E7/BLUE-LN/E4/E6/E8/BLUE-HN	10.24/6.04/12.39/12.11/9.53/9.38/12.3 **a** (10.30)			E5/E4/E6/BLUE-LN	6.37/8.51/4.33/7.70 **b** (6.73)
*QTkw3D.2*	AX-94785859-AX-108969398	522.08–555.08	E5/E10/BLUE-LN	3.52/3.22/4.25 (3.66)	E5/BLUE-LN/E6	9.05/5.28/5.26 **b** (6.53)			E5/E3/BLUE-LN/E6	5.19/3.21/4.90/5.20 **a** (4.63)
*QTkw4A.4*	AX-111591351-AX-94881516	563.91–589.91	E2/BLUE-HN	11.23/3.68 (7.46)					E2/E5/E5	11.58/7.91/5.88 **a** (8.46)
*QTkw4A.6*	AX-111201251-AX-111711476	624.58–657.91	E1/E4/BLUE-LN/E3/E6/E5	5.22/8.20/8.75/6.32/3.76/9.76 (7.00)	E3/E5/E4/BLUE-LN/E1	6.75/5.09/6.92/6.28/5.22 **a** (6.05)			E4/BLUE-LN/E3/E6/BLUE-HN	6.13/7.02/6.58/4.21/7.06 **a** (6.2)
*QTkw4A.8*	AX-89599752-AX-110614619	682.79–686.14	E1/E10	6.81/3.25 (5.03) **c**						
*QTkw4B.1*	AX-109357924-AX-108914451	15.7–35.70	E4/BLUE-HN/E10	4.51/20.43/22.43 (15.79)	E4/E6/BLUE-HN/E10	6.29/14.00/23.20/33.55 **b** (19.26)	E4/BLUE-HN/E10	5.43/4.69/11.19 **b** (7.1)	E4/BLUE-HN/E10/E6	5.11/14.69/16.98/8.04 **b** (11.21)
*QTkw4B.2*	AX-109909104-AX-109315850	65.70–76.70	E5	15.24	E2/E5	6.93/17.68 **b** (12.31)				
*QTkw4B.3*	AX-111593917-AX-110022862	144.70–178.70	E6/E2	21.82/5.16 (13.49)	E6/E4	8.86/7.90 **b** (8.38)				
*QTkw4B.4*	AX-110998404-AX-109473770	402.70–438.70	BLUE-LN	5.43	BLUE-LN/E1	9.25/10.62 **b** (9.94)	E6/E1	20.21/10.62 **b** (15.42)	E6/BLUE-LN	8.55/5.83 **b** (7.19)
*QTkw4D.2*	AX-108874010-AX-111072884	52.53–67.53	BLUE-HN/E6	3.35/4.34 (3.85)					E8/E6	4.84/6.58 **b** (5.71)
*QTkw5A.4*	AX-111479128-AX-111562392	551.56–557.79	E5/E6	2.57/3.92 (3.25)					E7/E4	4.71/5.27 **b** (4.99)
*QTkw5A.5*	AX-109524552-AX-109031208	601.85–602.95	E10	10.59					E10/BLUE-HN	3.95/3.90 **b**(3.93)
*QTkw5B.5*	AX-110926792-AX-110417642	594.56–595.62			E3/E4/E10	4.36/3.78/2.46 (3.53) **d**				
*QTkw5D.3*	AX-110391491-AX-89331886	429.78–438.42	BLUE-LN	2.90					BLUE-HN/E3	4.08/3.87 **b** (3.98)
*QTkw6A.1*	AX-109585639-AX-109979359	40.60–49.46							E9/E1	4.96/6.45 (5.70) **d**
*QTkw6A.3*	AX-111050302-AX-108817049	412.90–417.96	E9/BLUE-LN	6.21/6.09 (6.15)	E9/BLUE-LN	5.79/4.25 **b** (5.02)				
*QTkw6A.4*	AX-108849013-AX-109467855	561.01–574.01							BLUE-LN/E10	4.02/4.13 (4.08) **d**
*QTkw6B.1*	AX-109901945-AX-109871569	271.76–279.76	BLUE-HN/BLUE-LN	5.22/7.96 (6.59) **c**						
*QTkw6B.2*	AX-109968521-AX-108962284	453.76–457.76	E4/E3/E6	8.90/10.12/5.38 (8.13)	E4/E9/BLUE-LN/E3/E10	7.17/6.73/8.15/9.74/2.78 **b** (6.91)			E9/BLUE-LN/E3	8.17/5.90/5.97 **b** (6.68)
*QTkw6B.3*	AX-110149880-AX-110014652	514.73–515.88	E1/E9	7.03/7.11 (7.07) **c**						
*QTkw7A.1*	AX-108833379-AX-110397800	122.95–130.95					E2/BLUE-HN	7.64/4.23 (5.93) **d**		
*QTkw7A.2*	AX-110487560-AX-109420524	664.95–688.79	E8	9.2					E4/E8/BLUE-HN	5.30/9.61/3.76 **b** (6.22)

In the table, “a” represents the average PVE of the conditional QTL with no significant change (no more than 1%) compared to that of the unconditional one; “b” represents the average PVE of the conditional QTL with a significant change (more than 1%) compared to that of the unconditional one; “c” represents the unconditional QTLs that were not detected in the conditional QTL analysis; and “d” represents the QTLs that were additionally detected in the conditional analysis. The numbers in brackets represent the average PVE of QTLs detected in different environments.

**Table 4:** QTL analysis of dominant epistasis in the KJ-RIL population.

QTL ^a^	ENV ^b^	Position1 (Mb)	Flanking Markers	QTL	Position2 (Mb)	Flanking Markers	LOD	PVE% (AA) ^c^	AA ^d^
*QTkw3A.3*	E2	230.76	AX-109930806-AX-94495161	*QTkw4A.4*	561.91	AX-111591351-AX-94881516	3.24	9.29	1.08
*QTkw4A.4*	BLUE-HN	541.91	AX-111591351-AX-94881516	*QTkw5A.1*	400.67	AX-110393916-AX-108821970	3.27	9.97	0.90
*QKl3A.6*	E5	410.763	AX-109416489-AX-111049355	*QKl5D.2*	470.6189	AX-111175690-AX-109382782	3.28	9.55	0.16
*QKl4A.3*	E7	581.9098	AX-110526171-AX-111830901	*QKl6D.6*	400.4398	AX-108821954-AX-110689037	3.22	9.48	0.16
*QKw2B*	E10	50.06	AX-109364692-AX-111111325	*QKw4A.1*	71.91	AX-111163489-AX-111591351	3.1	7.74	0.1
*QKw2D.1*	E10	276.75	AX-108885026-AX-111837110	*QKw4A.3*	321.91	AX-111591351-AX-94881516	3.43	7.66	−0.09
*QKdr5B.1*	E4	310.3226	AX-94627936-AX-109846736	*QKdr7B.2*	720.0621	AX-109274013-AX-109295053	3.98	10.36	0.07

Letter “a” indicates the QTL of epistatic interactions; letter “b” indicates the environment in which the QTL was detected; letter “c” indicates the phenotypic variation explained by epistatic QTL; letter “d” indicates an additive × additive (AA) effect; positive values indicate that two QTLs with the same parental allele (“KN9204” or “J411”) produce a positive effect, while the negative values represent two QTLs with a negative effect.

## Data Availability

The data presented in this study are included in the Supplementary Materials.

## Supplementary Materials

The following supporting information can be downloaded at: https://www.mdpi.com/article/10.3390/plants14121848/s1.

## Abbreviations

The following abbreviations are used in this manuscript:

CV	Coefficient of Variation
KU	Kurtosis
KDR	Kernel Diameter Ratio
KL	Kernel Length
KW	Kernel Width
KYPP	Kernel Yield Per Plant
KNPS	Kernel Number Per Spike
PH	Plant Height
QTL	Quantitative Trait Loci
RIL	Recombinant Inbred Line
SD	Standard Deviation
SK	Skewness
SNPS	Spikelet Number Per Spike
SN	Spike Number
SL	Spike Length
TKW	Thousand-Kernel Weight
